# Two Decades of Contemporary Surgery of Primary Cardiac Tumors

**DOI:** 10.1055/s-0038-1673333

**Published:** 2018-10-16

**Authors:** Khalil Jawad, Tamer Owais, Stefan Feder, Sven Lehmann, Martin Misfeld, Jens Garbade, Michael Borger

**Affiliations:** 1 Department of Cardiac Surgery, Heart Center, University of Leipzig, Leipzig, Germany; 2 Department of Cardiac Surgery, Central Hospital Bad Berka, Bad Berka, Germany

**Keywords:** cardiac tumor, minimally invasive heart surgery, surgical outcome

## Abstract

**Objective** The decision to operate cardiac tumors is an issue of balancing surgical outcome and survival with quality of life (QOL). We report our single-center experience in managing primary cardiac tumors between 1994 and 2014.

**Methods and Results** In this study, 269 patients were subjected to our standardized operative protocols, preoperative preparations, postoperative follow-up, and consents of participation. Demographic and preoperative/intraoperative/postoperative variables were collected with focus on long-term follow-up and survival. A total of 72,000 cardiac procedures were performed within 20 years at our institution. Two hundred sixty-nine patients were diagnosed with primary cardiac tumors (0.37%), with a male:female ratio of 1:1.68, mean age of 57.4 ± 19.5 years, and body mass index of 25.49 ± 6.5. The most presenting symptoms were dyspnea ( *n* = 94), arrhythmias ( *n* = 53), embolic event ( *n* = 36), and chest pain ( *n* = 29), and 33 patients were accidentally discovered. Isolated tumor excision and concomitant ablation were performed on 181 patients, while the rest needed additional procedures such as coronary artery bypass grafting ( *n* = 27) or valve surgery ( *n* = 61). Focus on pathology, tumor location was done reporting the commonest pathology such as myxoma ( *n* = 177) and fibroelastoma ( *n* = 56). The frequent site was the left atrium ( *n* = 162). Our primary results showed incidence of bleeding in 9 patients (3.3%), arrhythmias in 76 patients (28.25%), and mortality in 49 patients (18.2%). Five patients (1.8%) showed recurrence and 220 patients (81.8%) showed complaint-free survival.

**Conclusion** Complete excision of primary cardiac tumors is the golden rule in management as it improves survival and decreases morbidity expected from the progressing tumors process. The progression of minimally invasive techniques improves QOL and should be performed whenever possible.

Primary cardiac tumors can be classified as either benign/malignant or primary/secondary of malignant pathology. Data from 22 large autopsy series reported by McAllister et al showed the frequency of primary cardiac tumors is ∼0.02%, corresponding to 200 tumors in 1 million autopsies. 1 Around 75% are benign; nearly half are myxomas; and a majority of the rest are lipomas, papillary fibroelastomas, and rhabdomyomas. 1 Sixty years ago in 1954, Crafoord 3 was the first to excise an atrial myxoma on cardiopulmonary bypass. 2 Today, cardiac tumors represent only 0.3% of all open-heart surgeries. 3 The advent of modern surgical techniques, such as minimally invasive approaches, transformed the concept of high-risk surgery in cardiac tumor morbid patients into a surgery of lower and accepted risk. However, surgery is always indicated especially if obstructing intracardial flow or causing valvular dysfunction or conduction errors. 4 This squeal denotes probably advanced tumorous stage, thereby limiting therapeutic options and expressing the benefits of minimally invasive approaches. 5 In this report, we review the clinical and surgical experience and results of cardiac tumors at our Department of Cardiac Surgery, University of Leipzig, over the past 20 years. Survivors were contacted via telephone. The mean follow-up for this series is 5.3 years (range: 0.003–19.98 years). Our focus in this study was relating the pathology, localization, symptoms, as well as age and gender distribution to the long-term results regarding survival, tumor relapse, and risk of recurrence matched to the corresponding neoplastic etiology using descriptive statistical measurements.

## Methods

### Data Collection

The medical records of the Leipzig Heart Center and the patient database of the Department of Cardiac Surgery were pro- and retrospectively reviewed to collect patients with primary cardiac tumors between 1994 and 2014. Approval to conduct this study was obtained by the Institutional Review Board of the University of Leipzig and patient consent was waived. All necessary detailed data concerning presentation, diagnosis, treatment, and follow-up were obtained. All patients with thrombus and tumors metastatic to the heart were excluded.

All survivors were contacted via telephone. For additional missing medical data, the family doctor in charge was contacted as well. Follow-up was performed for every patient. Survival data were completed with the nationwide statistical database, in which every death is recorded. The follow-up time was calculated either to death or to the last verified contact with the living patient. Median follow-up was 4.93 ± 3.95 years.

Treatment entailed complete medical–surgical protocols, beginning from only sternotomy or minimally invasive heart surgery tumor excision and/or adjuvant therapy for malignant tumors. After interdisciplinary discussion with the oncology department, adjuvant therapy for sarcoma patients was decided to be either a combination chemotherapy (six courses of ifosfamide [1,500 mg/m ^2^ , days 1–4], dacarbazine [200 mg/m ^2^ , days 1–4], and doxorubicin [25 mg/m ^2^ , days 1–2] administered in 14-day intervals and granulocyte colony-stimulating factor [30 × 10 ^6^ IU/d, subcutaneous] on days 5–13), or six courses of monochemotherapy (doxorubicin) and radiation, or six courses of monochemotherapy alone (doxorubicin or Herceptin), or radiation alone. Radiation dose was based on age, gender, and seize adjusted and ranged from a total dose of 15 to 60 Gy. Adjuvant therapy for our patients suffering from a non-Hodgkin's lymphoma (diffuse large B cell lymphoma) consisted of six courses of rituximab, cyclophosphamide, doxorubicin, vincristine, and prednisone. Of the 269 patients, 100 were males and 169 were females. The youngest patient was 7 days and the oldest was 87 years (mean age: 57.4). Age–gender correlation, when taking all tumor pathologies into account, denoted that women were statistically significantly older ( *p* = 0.02) than their male counterparts at the time of clinical presentation (61.6 ± 14.5 vs. 51.6 ± 18.7 years).

**Fig. 1:**
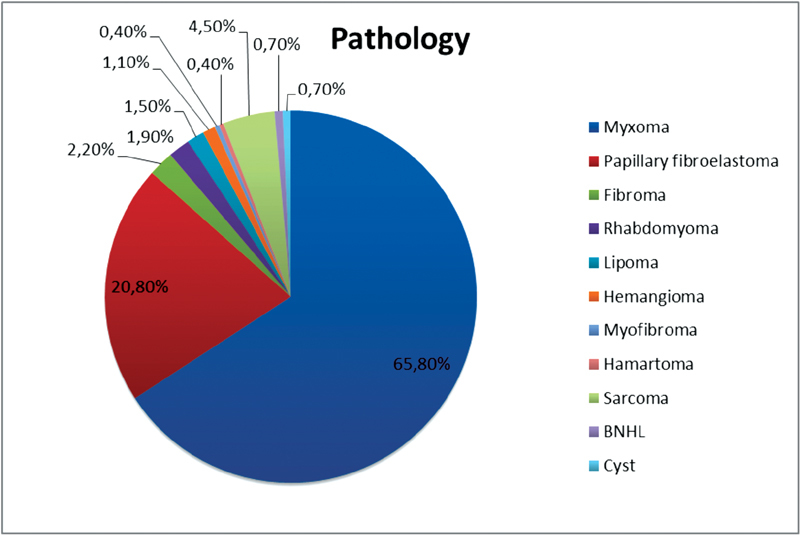
Distribution of cardiac tumors.

Tumor characteristics are summarized in Fig. 1 . Among our study group, 65.8% were diagnosed as myxomas and 20.8% as fibroelastomas. The commonest location was the left atrium among 162 patients (60.2%) and was mostly myxomas. The localization of fibroelastomas was more heterogeneous with a high affinity for aortic and mitral valve leaflets. Malignant cardiac tumors, whether sarcomas or lymphomas, composed a minor portion of our study population (14 patients [5.2%]). The complete demographic profile of all patients is presented in Table 1 .

**Table 1:** Baseline patient's characteristics

Variables	*n* (%)	Mean (SD)
Male	100 (37.2)	
Female	169 (62.8)	
Age (y)		
< 20	21 (7.8)	
20–40	16 (5.9)	
40–60	84 (31.2)	
60–80	142 (52.7)	
> 80	6 (2.2)	
BMI (kg/m ^2^ )		25.49 ± 6.5
Hypertension	161 (60.1)	
Diabetes	53 (19.8)	
Left ventricular function		
< 30%	11 (4.19)	
30–60%	119 (54.41)	
> 60%	132 (49.07)	
Presenting symptoms		
Dyspnea	34.9%	
Chest pain	10.8%	
Syncope	10%	
Emboli	13.4%	
Arrhythmia	21.2%	
Accidentally	12.3%	
Vegetative	18.6%	
Others	3.7%	

Abbreviations: BMI, body mass index; SD, standard deviation.

### Preoperative Evaluation

Related symptoms were dyspnea in 94 patients (34.9%), general fatigue in 50 patients (18.6%), central or peripheral neurological thromboembolic events in 36 patients (13.4%), and angina in 29 patients (10.8%). Subset of patients was asymptomatic and was accidentally discovered in 33 patients (12.3%), whereas another subset was presenting with symptoms of congestive heart failure (20.1%), who were in New York Heart Association (NYHA) class III or IV.

The presence of a tumor was diagnosed by echocardiogram. For morphological description in suspected cases, whether computed tomography or magnetic resonance imaging was performed. Coronary angiography was performed in all patients older than 40 years without risk factors (family history, dyslipidemia, and smoking) and in all patients older than 35 years in the presence of risk factors ( Table 2 ).

**Table 2:** Diagnostic

	*n* (%)
CT	3 (1.1)
Echo	139 (51.7)
Echo/Angio	30 (11.2)
Echo/Angio/CT	7 (2.6)
Echo/CT	28 (10.4)
Echo/CT/MRI	16 (5.9)
Echo/MRI	40 (14.9)
Echo/MRI/Angio	4 (1.5)
Echo/PET/CT	1 (0.4)
MRI	1 (0.4)
All	269 (100)

Abbreviations: Angio, angiography; CT, computed tomography; Echo, echocardiography; MRI, magnetic resonance imaging; PET, positron emission tomography.

### Operative Techniques and Evaluation

**Fig. 2:**
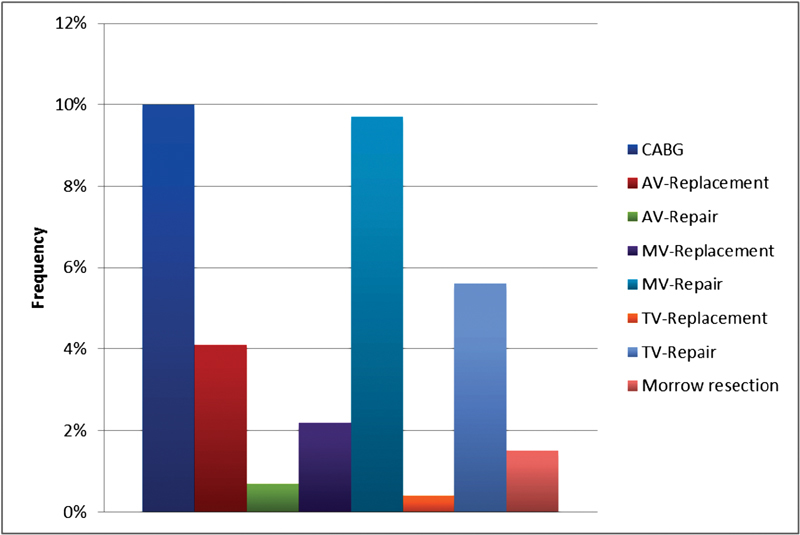
Concomitant procedure.AV, aortic valve; CABG, coronary artery bypass grafting; MV, mitral valve; TV, tricuspid valve.

Principally, patients with cardiac tumors were subjected to surgery because of the possibility of embolic complications or sudden death. According to the morphological picture of the tumor and its localization, we decided whether to perform a midline sternotomy or a minimally invasive approach (either upper ministernotomy or right-sided lateral thoracotomy). Conduction of cardiopulmonary bypass was through the routine cannulation sites and under moderate systemic hypothermia, deep topical hypothermia, and cardioplegic cardiac arrest. The average bypass time was 94.01 ± 206.2 minutes and ischemic time was 42.57 ± 30.8 minutes. Incisions were mostly performed through the left atriotomy (57.99%) and the second most common was the right atriotomy (28.99%). Rare incisions were aortotomy (9.29%), ventriculotomy (1.85%), and pulmonary arteriotomy (0.74%) according to the tumor localization. Detailed localization in the left atrium was not documented. Wide tumor excision with wide safety margins was our policy. In addition, cryoablation of the tumor base was done in cases where the tumor was showing deep intramyocardial invasion. Associated procedures included: coronary artery bypass grafting in 27 patients, mitral valve repair in 26 patients, mitral valve replacement in 6 patients, aortic valve replacement in 11 patients, aortic valve repair in 2 patients, tricuspid valve replacement in 1 patient, and repair in 15 patients ( Table 3 and Fig. 2 ).

**Table 3:** Operating data

	*n* (%)	Mean (SD)
Cross-clamp time (min)		42.57 ± 30.83
CPB time (min)		94.01 ± 206.29
Concomitant procedure		
CABG	27 (10)	
AV replacement	11 (4.1)	
AV repair	2 (0.7)	
MV replacement	6 (2.2)	
MV repair	26 (9.7)	
TV replacement	1 (0.4)	
TV repair	15 (5.6)	
Morrow resection	4 (1.5)	
Tumor location		
Left atrium	162 (60.2)	
Left ventricular	26 (9.7)	
Valves	37 (13.7)	
Right atrium	33 (12.3)	
Right ventricular	6 (2.2)	
Others	5 (1.9)	

Abbreviations: AV, aortic valve; CABG, coronary artery bypass grafting; CPB, cardiopulmonary bypass; MV, mitral valve; TV, tricuspid valve.

### Statistical Analysis

Standard definitions were used for patient variables and outcomes. Categorical variables were expressed as percentages and continuous variables as mean ± standard deviation (range). All statistical analyses were performed using IBM SPSS version 19.0 software (IBM Corp., New York). Comparisons of the preoperative and follow-up results were performed using a two-paired *t* -test and the Wilcoxon's signed rank test, respectively. Long-term survival of groups was evaluated and compared using the Kaplan–Meier's survival plot and the log-rank test, respectively. A two-sided *p* -value <0.05 was considered to be statistically significant.

## Results

**Fig. 3:**
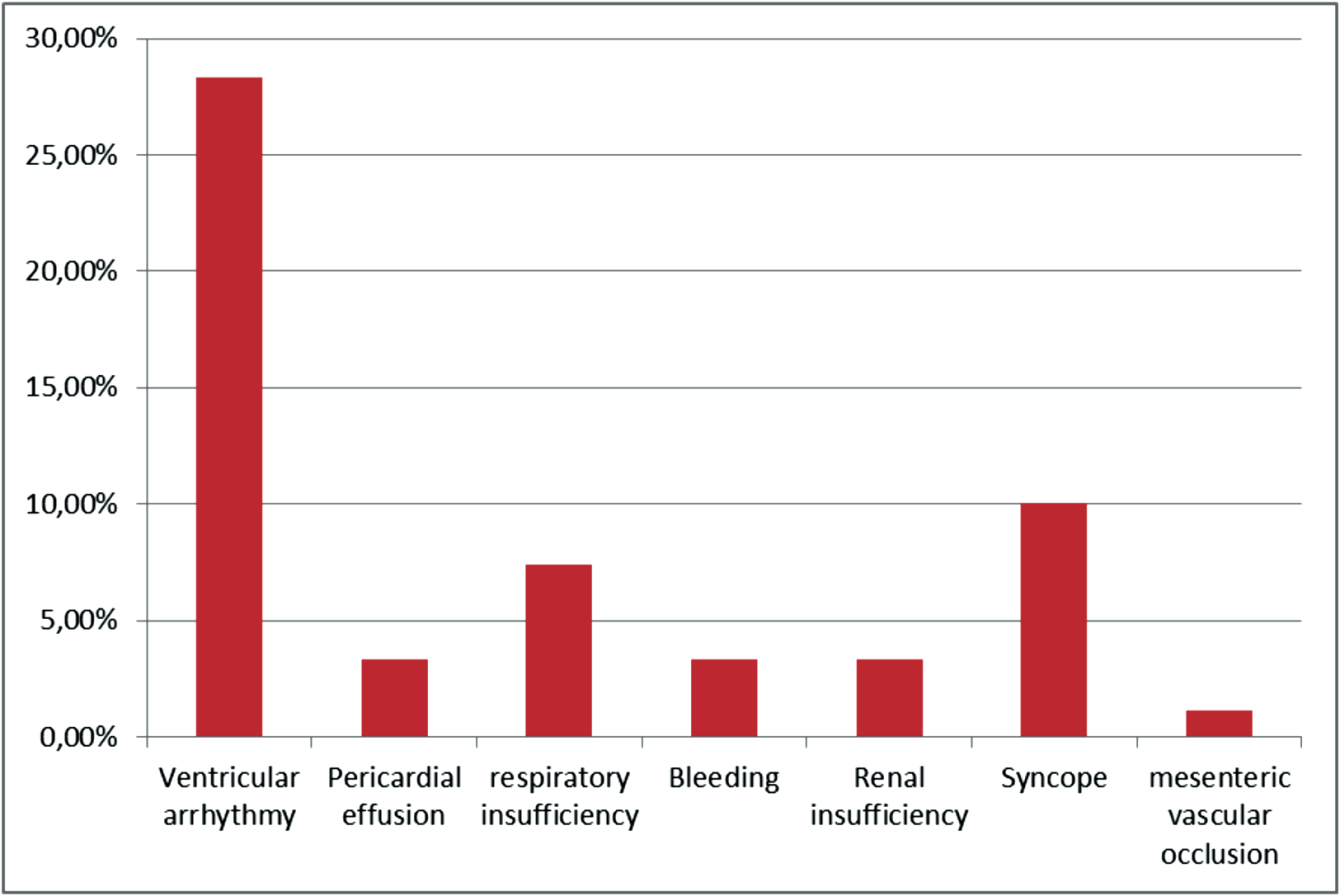
Postoperative complications.

**Fig. 4:**
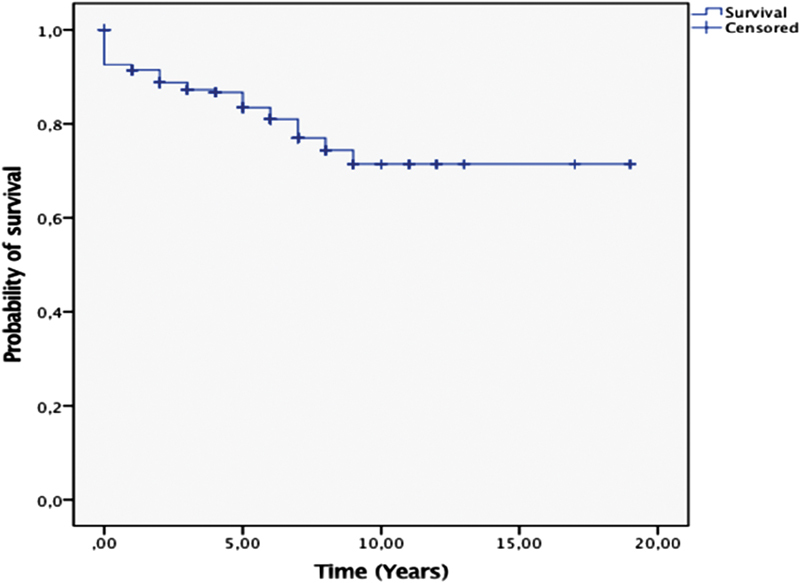
Kaplan–Meier's survival analysis: all tumors.

From 1994 to 2014, 72,000 cardiac procedures were performed at our institution. Among these procedures, a total of 269 patients were operated due to cardiac tumor masses (incidence 0.37%); 73% of the patients were women. Among our study group, 65.8% were diagnosed as myxomas and 20.8% as fibroelastomas. The commonest location was the left atrium among 162 patients (60.2%) and was mostly myxomas. The localization of fibroelastomas was more heterogeneous with a high affinity for aortic and mitral valve leaflets. According to the morphological picture of the tumor and its localization, we decided whether to perform a midline sternotomy (43.1%) or a minimally invasive approach (56.8%, either upper ministernotomy or right-sided lateral thoracotomy). Early postoperative results showed atrial or ventricular arrhythmias in 76 patients (28.3%), syncope in 27 patients (10.03%), respiratory insufficiency in 20 patients (7.4%), occurrence of bleeding in 9 patients (3.3%), renal insufficiency in 9 patients (3.3%), pericardial effusion also in 9 patients (3.3%), and mesenteric vascular occlusion in 3 patients (1.1%) ( Fig. 3 ). No intraoperative mortality was recorded. In-hospital mortality was 5 patients (1.9%) and mortality during follow-up was 49 patients (18.2%) with follow-up period ranged from 0.003 to 19.98 years ( Fig. 4 ). In our study, we correlated the incidence of survival with various parameters such as age, gender, pathology, preoperative NYHA class and ejection fraction, postoperative bleeding and arrhythmias, and finally tumor localization. Our follow-up and statistical analysis showed that age, pathology, and NYHA class are dependent factors affecting survival ( Table 4 ).

**Table 4:** Cox regression analysis

Variable	HR (95%CI)	*p* -Value
Age	2.320 (1.14–4.68)	0.019
Pathology	1.238 (1.15–1.33)	<0.001
NYHA	1.392 (1.02–1.9)	0.037

Abbreviations: CI, confidence interval; HR, hazard ratio; NYHA, New York Heart Association.

## Discussion

In our single-center long-term survey of patients with cardiac masses, we have shown a very low incidence of this disease with excellent surgical results and low morbidity and mortality.

Our age–gender correlation reported a statistically significant older age in females to males. This coincides with the findings of the University of Vienna in 2015. 6

It is worth noting that we did not exclude any patient from our study group based on gender. Our excluded patients were those with secondary metastasis and two patients with primary cardiac tumors, one of them was a male with heart failure and mechanically ventilated on high doses of vasopressors and the other was a female with a EuroSCORE of 39 and mechanically resuscitated on admission with persistent metabolic acidosis and nearly in a terminal clinical state.

Throughout our study group, we found that the commonest symptom was dyspnea (34.9%), followed by constitutional manifestations (18.6%) and the third commonest was accidental discovery of the tumor as well as emboli (13.4% and 12.3%, respectively). This does not coincide with the preoperative symptomatology stated by Dein et al 7 who stated dyspnea in 88%, neurological symptoms in 22%, and then 11% with constitutional manifestations. From our point of view, this lack of similarity returns to the strict and detailed preoperative work-up, and the experienced detailed intraoperative echocardiography, which is particularly helpful and specific. 8 This enabled us to accidentally discover many cases and upgraded this domain as the fourth commonest symptom. We did not focus on pathology-related symptomatology in our study; however, it is already thoroughly discussed in the literature that tumors of malignant origin usually present more aggressively than benign ones. 6

Choice of the operative technique and incision is mainly based on tumor localization, morphology, expected pathology, and surgical experience. We tended more toward minimally invasive approach whenever possible. In our experience, after the advent of transesophageal echocardiography, we believe visual inspection of all four cardiac chambers is not necessary. Although Jones et al recommended a sternotomy biatrial approach to allow minimal manipulation of the tumor, provided adequate exposure for complete resection, allow inspection of all four heart chambers, and minimize recurrence. 9 We were able to reach the four goals of Jones et al throughout using a minimally invasive approach. Most of these minimally invasive patients were mainly addressed to right or left atriotomies, but unfortunately, we recorded conversion to sternotomy between two patients (0.7%) because of surgical surprise in the sense of highly invading inaccessible tumors which were not accurately seen through the preoperative imaging tools. Of note, those conversion patients had pathologically papillary fibroelastoma tumor. Some authors also reported low recurrence and safety of these incisions (right or left atriotomy) as well. 4 5

There is still considerable controversy concerning the extent of resection, usage of patch repairs, or endocardial tumor base ablation. Our policy was to excise until the base but preserving the endomyocardial and intramyocardial thickness then substituting the extended base resection with endocardial ablation. Our results were satisfactory in this domain, recording lower bypass times (mean 94.01 minutes), ischemic times (mean 42.5 minutes), and lower recurrence in five patients (1.85%). On the contrary, other satisfactory results were reached through only wide excision and patch repair 10 and some others through only simple excision recording recurrence-free periods of 4.5 to 10 years. 11 12 In our study, septal defects were repaired usually with bovine pericardial patch. Only one of the five patients needed a septal patch repair. In two patients, a primary repair was sufficient.

Recurrence could be related to various factors such as the primary pathology and extent of resection. 13 However, it is advised for rapidly growing tumors to be excised with a wide safety margin, and for benign local tumors such as myxomas, it is not always necessary to completely resect the septum or atrial wall. Our recurrence results are better than some authors who found out 5% recurrence of myxomas 14 after extended resection. In comparison, there was 1.85% recurrence in our study. We argue this fact for the addition of endocardial ablation in the base of the tumors as routine step after excision. Moreover, this adds to the theory that minimally invasive approaches are not inferior to sternotomy concerning recurrence rates, since 153 patients (56.8%) from our study were operated on using this technique.

Our survival rates were studied in correlation to all variables to try to get independent factors of survival. Concerning gender-related survival, we found there were more female survivals than males in the first 7 follow-up years. Beginning from the seventh year, the male survivals were more than females. This could be explained through the fact that our age–gender preoperative correlation showed a highly advanced age in the female group to the male group. We concluded statistically significant higher survival among patients, who were operated upon at age younger than 63 years. Despite this finding, we cannot define an age limit above which surgery is contraindicated because surgical excision remains the main curative modality irrespective of age. Only in special cases do we have to weigh operability, survival, and life quality with age.

**Fig. 5:**
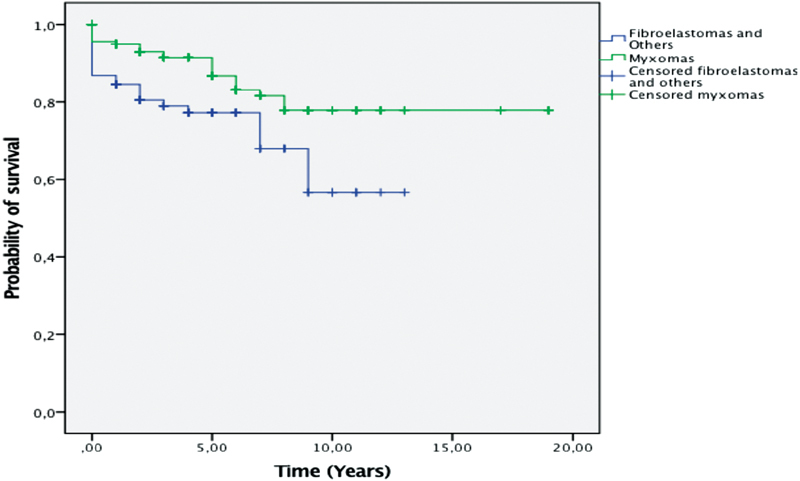
Kaplan–Meier's survival analysis: myxomas versus others.

Pathology-related survival showed relative equal survival between myxomas and fibroelastomas in the first 7 years of follow-up. After that period, myxoma patients showed statistically significant higher survival log rank ( *p* < 0.01) ( Fig. 5 ).

Our results showed significantly lower survivals among sarcomas and lymphomas. This coincides with Marvasti et al 15 concerning myxomas survivals and Poole et al concerning malignancy survivals. 16 Our explanation of this fact was that the relative ease of technical feasibility of tumor resection had a significant effect on mean patient survival following surgery. Syncope, as a preoperative symptom had no statistical significance on survival with a log rank ( *p* = 0.89). This could be explained through being the fourth commonest presentation among our study population. That is why we could not record any statistical significance in this domain. Survival statistically significant results concerning the NYHA class preoperatively were detected among our study population scoring a log rank of 0.00. Patients with NYHA IV preoperatively showed least survivals at 12 postoperative years of follow-up, while patients with no dyspnea as a presenting symptom recorded two plateau phases of stable survival rate, from years 1 to 7 and from year 10 until the end of follow-up. This fact was not studied in detail throughout the published literature in this domain as a result we could not find any comparative results concerning the NYHA-survival relation. The influence of preoperative ejection fraction on survival was also one of our focus points. Our results state statistically insignificant values of log rank 0.149. Localization of the cardiac tumor and postoperative arrhythmias was thoroughly studied and recorded statistically significant results with log rank 0.01 in both variables. We have relatively lower incidence of postoperative arrhythmias when compared with other publications. 17 This may be because of the tumor base cryoablation that we applied as routine step after tumor excision. Being a statistically significant parameter, we see that it is worth to further intensively investigate this as a separate parameter, although throughout our study it had no influence on survival. Finally, bleeding as a postoperative parameter was neither statistically significant nor of influence on survival.

Surgical resection, when possible, is the treatment of choice for all patients with cardiac neoplasms. The results of surgical treatment and survival are closely related and dependent on pathology, age, NYHA preoperatively, tumor localization, and postoperative arrhythmias of the tumors. Specifically, age, pathology, and NYHA preoperatively are dependent factors of survival. A close postoperative long-term follow-up and observation of these patients are recommended. It is curative in benign tumors and may prolong life in malignant tumors, although we think that development of minimally invasive techniques adds for improvement of life quality.
